# Enhancing and stabilizing monoclonal antibody production by Chinese hamster ovary (CHO) cells with optimized perfusion culture strategies

**DOI:** 10.3389/fbioe.2023.1112349

**Published:** 2023-01-20

**Authors:** Kexue Liang, Hongzhen Luo, Qi Li

**Affiliations:** ^1^ Key Laboratory of Industrial Biotechnology, Ministry of Education, School of Biotechnology, Jiangnan University, Wuxi, China; ^2^ School of Life Science and Food Engineering, Huaiyin Institute of Technology, Huaian, China

**Keywords:** perfusion culture, process optimization, Chinese hamster ovary cells, monoclonal antibody, product quality

## Abstract

The perfusion medium is critical in maintaining high cell concentration in cultures for the production of monoclonal antibody by Chinese hamster ovary cells. In this study, the effects of perfusion culture strategies when using different media on the process stability, product titer, and product quality were investigated in 3-L bioreactor. The results indicated that continuous perfusion could maintain higher levels of cell density, product titer, and quality in comparison with those of the intermittent perfusion culture. Next, the perfusion culture conditions with different perfusion rates and temperature reduction methods were further optimized. When combining the high perfusion rates and delayed reduction of culture temperature at day 6, the product titer reached a higher level of 16.19 g/L with the monomer relative abundant of 97.6%. In this case, the main peak of the product reached 56.3% and the total N-glycans ratio was 95.2%. To verify the effectiveness of the optimized perfusion culture in a larger scale, a 200-L bioreactor was used to perform and the final product titer reached the highest level of 16.79 g/L at day 16. Meanwhile, the product quality (monomer abundant of 97.6%, main peak of 56.3%, and N-glycans ratio of 96.5%) could also be well maintained. This study provided some guidance for the high-efficient production of monoclonal antibody by CHO cells *via* optimized perfusion culture strategy.

## 1 Introduction

Recently, monoclonal antibodies (mAbs) and recombinant biopharmaceutical proteins (rBPs) have revolutionized the pharmaceutical industry (Dahodwala and Lee 2019; Zhang et al., 2021). Since 2016, about 70% of all rBPs and mAbs were produced from Chinese hamster ovary (CHO) cell lines, which are attributed to their robust growth and potential to produce non-immunogenic antibodies with similar glycosylation patterns to those of human antibodies (Lalonde and Durocher 2017; Dahodwala and Lee 2019; MacDonald et al., 2021). Meanwhile, at the current approval rate of four new products a year, more than 70 mAbs were on the market by 2020, and the world-wide sales will reach $125 billion (Ecker et al., 2015; Bhatti and Salama 2018). Compared with most of the small molecular drugs, mAbs feature with some advantages such as reduced off-target effects, greater surface area for binding, etc. (Shepard et al., 2017; Ha et al., 2022). In order to improve the competitiveness of one mAb product, the mAb titer needs to be improved to elevate the economic effectiveness (Ha et al., 2022). Therefore, it is essential to develop advanced technology by process engineering, optimization, and control strategies.

Nowadays, fed-batch and perfusion cell cultures are the two current processes of the large-scale industrial production of mAbs and rBPs (Zhuang et al., 2017; Zheng et al., 2018; Schulze et al., 2022). Generally, scaling of the fed-batch process needs larger and more rigid layouts which limits its application (Karst et al., 2016; MacDonald et al., 2021). The membrane-based alternating tangential flow filtration (ATF) technology is the most commonly used cell retention method in perfusion cultures to increase cells density and mAbs productivity (Genzel et al., 2014; Gränicher et al., 2020). It has been reported that for the production of unstable therapeutic proteins, such as recombinant enzymes and blood coagulation factors, the perfusion process possesses more advantages (Bettinardi et al., 2020; MacDonald et al., 2021). In the perfusion mode, culture broth contained products and waste is perfused through the bioreactor under the perfusion controller, while the cells/products are retained or recycled back into the bioreactor (Ahn et al., 2008; MacDonald et al., 2021; MacDonald et al., 2022). Consequently, a consistent and steady culture condition with metabolized by-product rapidly removal results in a stable and uniform product of high quality under perfusion culture process (Schwarz et al., 2020; Yin et al., 2021). In general, culture environmental parameters including temperature, osmolality, levels of dissolved oxygen (DO), CO_2_ partial pressure (pCO_2_), and perfusion rate could affect the performance of recombinant protein production in CHO cells (Sou et al., 2017; Wang Q. et al., 2018b; Hippach et al., 2018; Madabhushi et al., 2021). Some studies found that the specific productivity of recombinant CHO cell lines and the product quality can be improved by lowering the culture temperature (Tait et al., 2013; Wang K. et al., 2018a). Due to the various quality attributes and types of proteins, the culture temperature and other environmental factors still should be optimized to improve the process stability and product quality when exploring a specified mAb.

Besides the improvement of product titer, consistently good product quality is also a considerable factor (Ha et al., 2022). The product quality is the suitability of a drug product for its intended use. Based on the current good manufacturing practices (cGMP) of the Food and Drug Administration (FDA) of U.S., the critical quality attributes include aggregation, charge variation, and glycosylation (Bonaccorso et al., 2021; Ha et al., 2022). In addition, scaling up the production process in a larger bioreactor for verifying the effectiveness of process strategy is the crucial step for the commercial category (Xing et al., 2009; Vodopivec et al., 2019).

Based on the above analysis, the objective of this study was to optimize the production process under perfusion culture strategy of the CHO cell line producing one anti-PD-1 mAb. Firstly, the effects of different media on the perfusion culture and product quality were investigated to reduce the raw material costs. Subsequently, the perfusion culture conditions, including perfusion rates and culture temperature, were further optimized for improving the product titer and quality. Finally, the effectiveness of the optimized perfusion culture strategy was successfully verified in a 200-L bioreactor. The obtained results could provide some guidance for the mAb production *via* process optimization and control methods.

## 2 Materials and methods

### 2.1 Cell line and culture media

The CHO cell line, which could produce anti-PD-1 mAb, was used in this work. Here, several media were chosen for CHO cells culture. The chemical composition of basic medium I (BM-I) was 24.30 g/kg Dynamics serum-free medium (Thermo Fisher, NH, United States), 15.49 g/kg hypoxanthine monosodium, 3.80 mg/kg thymidine, 0.57 g/kg glutamine, 0.70 g/kg cell boost 5 (HyClone^TM^), and 1.00 g/kg poloxamer 188 (HyClone^TM^). The feed medium I (FM-I) consists of 79.60 g/kg CD efficient feed C AGT nutrient supplement (Life Technologies Corporation, United States), 15.00 g/kg sheff-CHO plus PF ACF (Kerry Biofunctional Ingredients Inc., United States), and 13.30 g/kg 10 N NaOH solution. The feed medium II (FM-II) consists of 4.75 g/kg L-tyrosine, and 7.95 g/kg L-cystine. The chemical composition of basic medium II (BM-II) was 24.30 g/kg CD CHO AGT (Thermo Fisher, NH, United States), 0.58 g/kg glutamine, 15.81 mg/kg hypoxanthine monosodium, and 3.88 mg/kg thymidine. The basic medium III (BM-III) consists of 22.23 g/kg Eden B600S, 0.58 g/kg glutamine, 15.49 mg/kg hypoxanthine monosodium, 3.80 mg/kg thymidine, and 2.13 g/kg NaHCO_3_. The feed medium III (FM-III) consists of 177.05 g/kg Eden F600aS. The feed medium IV (FM-IV) consists of 71.84 g/kg Eden F600bS and 25.70 g/kg NaOH. The basic medium IV (BM-IV) consists of 22.31 g/kg Eden B501S, 0.58 g/kg glutamine, 15.49 mg/kg hypoxanthine monosodium, 3.80 mg/kg thymidine, and 2.18 g/kg NaHCO_3_. The feed medium V (FM-V) consists of 179.69 g/kg Eden F500aS. The feed medium VI (FM-VI) consists of 77.47 g/kg Eden F200bS and 29.05 g/kg NaOH. The feed medium VII (FM-VII) consists of 20.01 g/kg Eden B600S, 0.52 g/kg glutamine, 13.49 mg/kg hypoxanthine monosodium, 3.42 mg/kg thymidine, 1.92 g/kg NaHCO_3_, and 17.71 g/kg Eden F600aS. The commercial media including Eden B600S, Eden F600aS, F600bS, Eden B501S, Eden F500aS, Eden F200bS, and Eden B501S were purchased from Bio-Engine Co., Ltd., Shanghai, China.

### 2.2 Culture conditions

**FIGURE 1:**
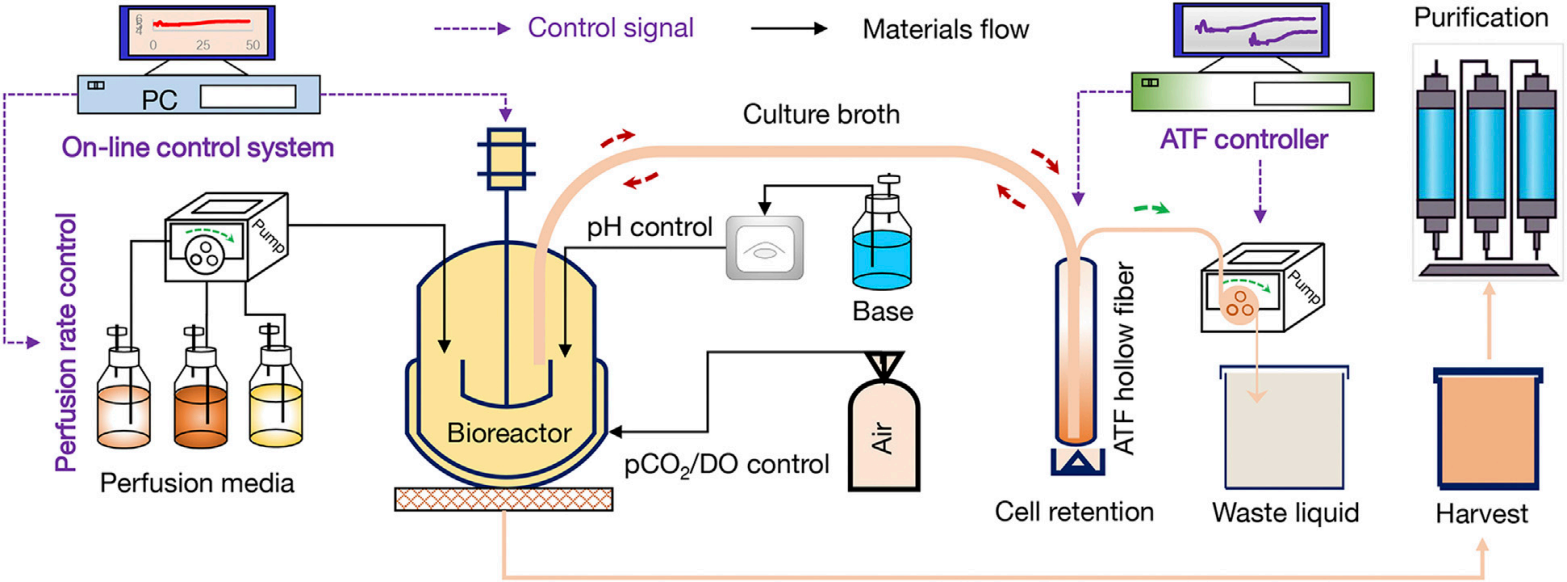
Schematic diagram of the CHO perfusion culture for the production of anti-PD-1 monoclonal antibody (mAb).

In this study, eight runs (#1-#8) were performed by using 3-L bioreactor (Applikon, Schiedam, Netherlands) for the optimization of perfusion culture strategy to produce anti-PD-1 mAb. Furthermore, run #9 was performed by using a 200-L bioreactor (Applikon, Schiedam, Netherlands) for verifying the effectiveness of perfusion culture for mAb production with a larger scale. The alternating tangential flow filtration (ATF) technology was used to develop a concentration culture by using the hollow fiber membranes (50 kDa). The schematic diagram of the CHO perfusion culture for the production of anti-PD-1 mAb was shown in Figure 1. In the perfusion cultures of runs #1-#8, 2 L of CHO cells with the density of 1.5×10^6^ cells/mL were inoculated in the 3-L bioreactor. For the run #9, 180 L of CHO cells with the density of 1.5×10^6^ cells/mL were inoculated in the 200-L bioreactor. The culture pH was automatically controlled at 6.80–6.90 by adding either NaHCO_3_ or CO_2_. The agitation speed was kept constant at 250 rpm during the whole culture process. The dissolved oxygen (DO) concentration was maintained at 40 ± 15% of air saturation. The initial culture temperature was controlled at 36.5°C during the growth stage and then decreased to 31.0°C at day 5 (runs #1-#4, run #5, and run #7) or day 6 (run #6 and runs #8-#9). The cultures were ended at day 14 or day 16. The perfusion rates (L/L/day, VVD) and culture conditions of runs #1-#8 were set as shown in Tables 1, 2. For the continuous perfusions (runs #1-#2, runs #4-#9), the ATF controller was started at day 2 and cells were pumped in and out of the hollow fiber with a setting rate by using different feeding media (Tables 1, 2). For the intermittent perfusion of run #3, the ATF controller was started at day 3 for running 24 h and then stopped for 24 h (the detailed perfusion rates were shown in Table 1). It should be noted that the run #5 was the perfusion culture with N-2/N-1 adaption by using BM-II as the basic medium. In addition, the perfusion rates and culture conditions of run #9 were the same as the run #8. A 50-mL sample was collected daily to determine viable cell density (VCD), viability, and the supernatant obtained after centrifugation was kept at −80°C for the further analysis of glucose concentration and lactate concentration.

**TABLE 1:** The perfusion rates (VVD) of perfusion media in runs #1-#4 by using 3-L bioreactor.

Culture time (day)	Run #1 (continuous perfusion)			Run #2 (continuous perfusion)			Run #3 (intermittent perfusion)			Run #4 (continuous perfusion)		
	BM-I	FM-I	FM-II	BM-II	FM-III	FM-IV	BM-II	FM-III	FM-IV	BM-IV	FM-V	FM-VI
D2	0.400	0.000	0.005	0.400	0.000	0.005	N/A	N/A	N/A	0.400	0.000	0.005
D3	0.450	0.050	0.005	0.450	0.050	0.005	0.500	0.050	0.005	0.450	0.050	0.005
D4	0.595	0.105	0.005	0.595	0.105	0.005	N/A	N/A	N/A	0.595	0.105	0.005
D5	0.680	0.120	0.005	0.680	0.120	0.005	0.500	0.050	0.005	0.680	0.120	0.005
D6	0.680	0.120	0.005	0.680	0.120	0.005	N/A	N/A	N/A	0.680	0.120	0.005
D7, D9, D11, D13	0.900	0.100	0.005	0.900	0.100	0.005	0.500	0.050	0.005	0.900	0.100	0.005
D8, D10, D12	0.900	0.100	0.005	0.900	0.100	0.005	N/A	N/A	N/A	0.900	0.100	0.005

Note: The culture temperature of runs #1-#4 was reduced at day 5.

**TABLE 2:** The optimization of perfusion culture in runs #5-#8 by using 3-L bioreactor.

Culture time (day)	Run #5			Run #6			Run #7			Run #8		
	BM-II	FM-III	FM-IV	BM-II	FM-III	FM-IV	BM-II	FM-III	FM-IV	BM-II	FM-III	FM-IV
D2	0.400	0.000	0.005	0.400	0.000	0.005	0.450	0.050	0.005	0.300	0.300	0.005
D3	0.450	0.050	0.005	0.450	0.050	0.005	0.450	0.050	0.005	0.585	0.065	0.005
D4	0.595	0.105	0.005	0.595	0.105	0.005	0.675	0.075	0.005	1.000	0.100	0.005
D5	0.680	0.120	0.005	0.680	0.120	0.005	0.675	0.075	0.005	1.000	0.100	0.005
D6	0.680	0.120	0.005	0.680	0.120	0.005	0.675	0.075	0.005	1.000	0.100	0.005
D7-D12	0.900	0.100	0.005	0.900	0.100	0.005	0.900	0.100	0.005	1.000	0.100	0.005
D13-D15	0.900	0.100	0.005	0.900	0.100	0.005	0.900	0.100	0.005	1.300	0.140	0.005

Note: The culture temperature of run #5 and run #7 was reduced at day 5. Run #5 refers to the perfusion culture with N-2/N-1 adaption by using BM-II as the basic medium. Run #6 refers to the perfusion culture with the same perfusion rates, while the temperature was reduced at day 6. Run #7 refers to the perfusion culture with different perfusion rates of run #5-#6. Run #8 refers to the perfusion culture with higher perfusion rates and the temperature was reduced at day 6.

### 2.3 Analytical methods

CHO cells were sampled from bioreactor every day, and the cell density and viability were then measured with an automatic cell counter (Vi-Cell XR, Beckham Coulter Inc., United States). The mAb titer was measured by a POROS™ A20 column (2.1 mm × 30 mm, Thermo Fisher Scientific) with an HPLC system (Agilent 1260 Infinity II). Antibody purity and aggregation analysis were performed by the analytical size-exclusive chromatography (SEC-HPLC, Agilent 1260, United States) with the column of TSKgel G3000SWXL (7.8 mm × 250 mm × 5 μm, Tosoh, Tokyo, Japan) (Zhuang et al., 2017). The charge variation of the anti-PD-1 mAb was determined by cation-exchange chromatography (CEX-HPLC). Antibody charge variants were separated on a column of ProPac WCX-10 (4 mm × 250 mm × 10 μm, Thermo Fisher Scientific, Sunnyvale, CA, United States) using an Agilent 1260 HPLC system at a flow rate of 1.0 mL/min (Zhuang et al., 2017).

The oligosaccharide profiles were ascertained for the quantitative assessment of the mAb products by an HPLC-based method (Zheng et al., 2018). For simplicity, the four dominant glycoforms are written as G0F, G1F, G2F, and Man-5, in which the letter “G” indicates glycans, the number signifies how many terminal galactose molecules are attached to the two arms of the glycan, and the letter “F” indicates a fucosylated N-linked glycan in the immunoglobulin G (IgG) Fc domain.

### 2.4 Statistical analysis

In this study, to ensure the data accuracy and reproducibility, all analytical methods used were validated prior to the measurements. The data are represented as mean ± SD of three biological independent experiments. Significant differences between perfusion cultures were confirmed by a two-tailed Student’s t-test using Microsoft Excel 2016. The statistical significance was considered as *p* < 0.05.

## 3 Results and discussion

### 3.1 Production performance of mAb under CHO cell perfusion cultures using different media in 3-L bioreactor

**FIGURE 2:**
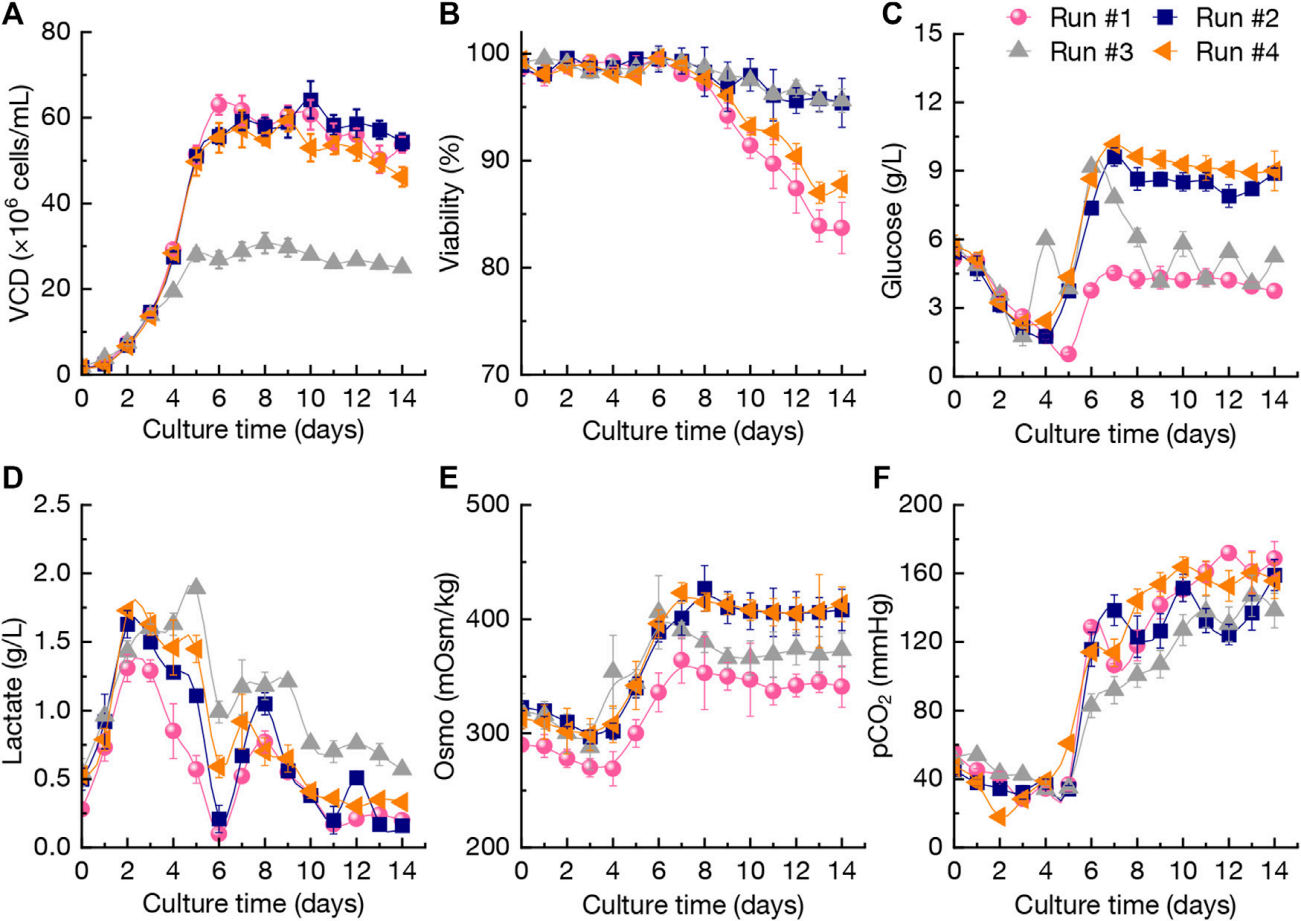
Changing profiles of viable cell density (VCD) **(A)**, viability **(B)**, glucose concentration **(C)**, lactate concentration **(D)**, osmolality (Osmo) **(E)**, and pCO_2_ **(F)** during perfusion cultures using different media in 3-L bioreactor. The error bars indicate the standard deviations (SD) of three independent experiments. Continuous perfusion: run #1, #2, and #4; intermittent perfusion: run #3. The perfusion rates and conditions in the four runs were shown in Table 1.

For the production of mAb using CHO cells, the raw materials cost (mainly the cost of culture medium) generally occupies high ratio over the total production cost. Therefore, several cultures using relative low-price medium to replace the high-price and commercial media (BM-I, FM-I, and FM-II used in run #1 as the control) were performed in a 3-L bioreactor to improve the overall economics. In this study, some culture media purchased from Bio-Engine Co., Ltd. (Shanghai, China) with low-prices were selected as the perfusion culture media for evaluation of the production performance of anti-PD-1 mAb. The perfusion culture conditions for runs #1-#4 were summarized in Table 1. The changing patterns of VCD, viability, glucose concentration, lactate concentration, osmolality (Osmo), and pCO_2_ during perfusion cultures by using different media in 3-L bioreactor were shown in Figure 2. The VCD during the first 3 days in runs #1-#4 stayed at the similar levels of 1.5 × 10^6^–17×10^6^ cells/mL although using different media for perfusion culture (Figure 2A). When using BM-I, FM-I, and FM-II as the media for continuous perfusion (run #1), the highest VCD reached 63.0×10^6^ cells/mL at day 6, while it then gradually decreased to a final level of 53.7×10^6^ cells/mL at day 14. In run #2, the alternative media of BM-II, FM-III, and FM-IV were used for continuous perfusion after day 2 and the VCD during day 5–14 was kept at 50.1 × 10^6^–65.6×10^6^ cells/mL (Figure 2A). In addition, the intermittent perfusion culture when using BM-II, FM-III, and FM-IV was also performed (run #3). The VCD data of run #3 showed that intermittent perfusion could not elevate the cells density, which is mainly attributed to the nutrient shortage (Pérez-Rodriguez et al., 2020; MacDonald et al., 2021). The results indicated that continuous perfusion could maintain higher VCD levels (Figure 2A). As shown in Figure 2B, the viability of CHO cells during culture from day 0 to day 8 in four runs was maintained at > 95%, and it still kept at the similar levels in runs #2-#3 which used BM-II, FM-III, and FM-IV during the perfusion culture. In run #1 and run #4, the final viability of CHO cells was decreased to ∼85%. The above results of VCD and viability of CHO cells in the four runs revealed that BM-II, FM-III, and FM-IV were the better media for perfusion culture.

Besides CHO cells growth, the key metabolites including glucose and lactate in culture broth were also analyzed. The glucose concentration during the period of day 6–14 in run #1 ranged 3.73–4.53 g/L, which was lower than that of runs #2-#4 (Figure 2C). As shown in Figure 2D, the lactate concentration in run #3 under intermittent perfusion culture kept at the higher levels of 0.57–1.60 g/L after day 4. In addition, the lactate concentration in culture broth (runs #1, #2, and #4) was relative lower with a final accumulation of 0.2–0.3 g/L. It was reported that lactate accumulation during cell culture could cause negative effects on CHO cells growth and mAb production (Konakovsky et al., 2016; Graham et al., 2019), which could also be reflected by the VCD changing patterns in run #3. For the anti-CD52 rCHO cell perfusion cultures, it was found that the initial osmolality of 180 mOsm/kg in early stage of perfusion cultures could inhibit the viable cell density, but the stable osmolality finally achieves higher mAb production (Qin et al., 2019). As shown in Figure 2E, the osmolality changings in four runs revealed that the range of 300–400 mOsm/kg could not significantly inhibit the CHO cells growth. Furthermore, the pCO_2_ levels were shown in Figure 2F with no significant difference among different perfusion cultures.

**FIGURE 3:**
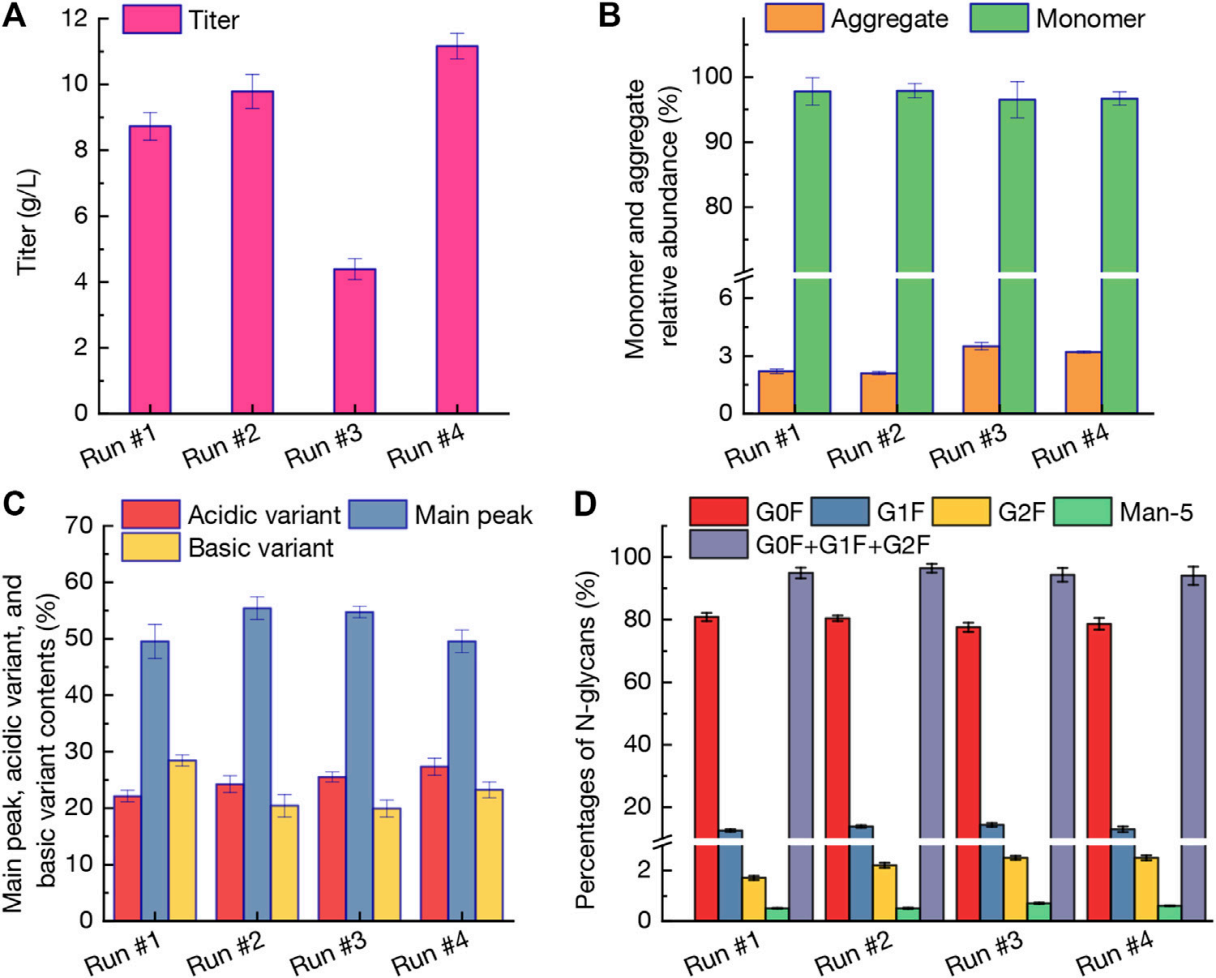
Profiles of mAb titer **(A)**, monomer and aggregate relative abundance **(B)**, variant contents **(C)**, and percentages of N-glycans **(D)** under perfusion cultures of run #1-#4. The error bars indicate the SD of three independent experiments.

The profiles of mAb titer, monomer and aggregate relative abundance, variant contents, and percentages of N-glycans under perfusion cultures of run #1-#4 were displayed in Figure 3. Obviously, under the intermittent perfusion culture strategy, the mAb production in run #3 was only 4.39 g/L at day 14, while the titer still reached to higher levels of 8.73–9.83 g/L in other three runs under continuous perfusion cultures (Figure 3A). The balance between light chains and heavy chains of immunoglobulins could change the aggregation of mAbs which is a crucial key in the mAb production process (van der Kant et al., 2017). As shown in Figure 3B, the monomer relative abundance in different runs was comparable which ranged from 96.5% to 97.9%. The aggregate relative abundance in mAbs obtained from intermittent perfusion culture (run #3) reached the highest level of 3.5% (vs. 2.1%–2.2% under continuous perfusion cultures of runs #1-#2). The results revealed that intermittent perfusion strategy could not only affect the CHO cells growth (Figure 2A), but also improve the aggregate relative abundance (Figure 3B) because of the varied growth conditions (Kim et al., 2008; Bettinardi et al., 2020). Due to the potential effects on the stability and biological potency, the charge heterogeneity is considered as the critical quality index during the mAb production processes (Hintersteiner et al., 2016; Qin et al., 2019). As shown in Figure 3C, the main peak contents of 55.4% in run #2 were significantly higher than those of run #1 (49.5%) and run #4 (49.5%). In addition, the acidic variant content in run #4 when using BM-IV, FM-V, and FM-VI as the perfusion media reached 27.5% with significant differences between other cases (*p* < 0.05, Figure 3C). The basic variant content in run #1 by using BM-I, FM-I, and FM-II as the perfusion media was 28.4% which would decrease the mAb quality. The N-linked oligosaccharides (i.e., N-glycans) profiles were shown in Figure 3D and four dominant glycoforms (G0F, G1F, G2F, and Man-5) were exhibited. There is no statistically significant difference for all glycoforms except for G1F and G2F. The Man-5 ratio in mAbs produced from different cultures was maintained 0.5%–0.7% which could meet the specified requirement for mAbs quality commercially (lower than 5.0%, Figure 3D). In summary, based on the above results including process parameters, anti-PD-1 mAb titer, and mAb quality, the continuous perfusion culture by using BM-II, FM-III, and FM-IV as the media (run #2) could replace the commercial media used in run #1 for mAb production.

### 3.2 Optimized CHO perfusion cultures for enhancing and stabilizing mAb production in 3-L bioreactor

In order to improve the overall performance of mAb production, several cultures of runs #5-#8 were further performed, and the culture conditions/perfusion rates were summarized in Table 2. In run #5, BM-II was used for the adaption of N-2/N-1 CHO cells for cultivation. In addition, the culture temperature was reduced at day 5 in runs #5 and #7, while it reduced at day 6 in runs #6 and #8. The perfusion rates were systematically optimized for evaluation of the effects on process parameters, mAb titers, and product quality.

**FIGURE 4:**
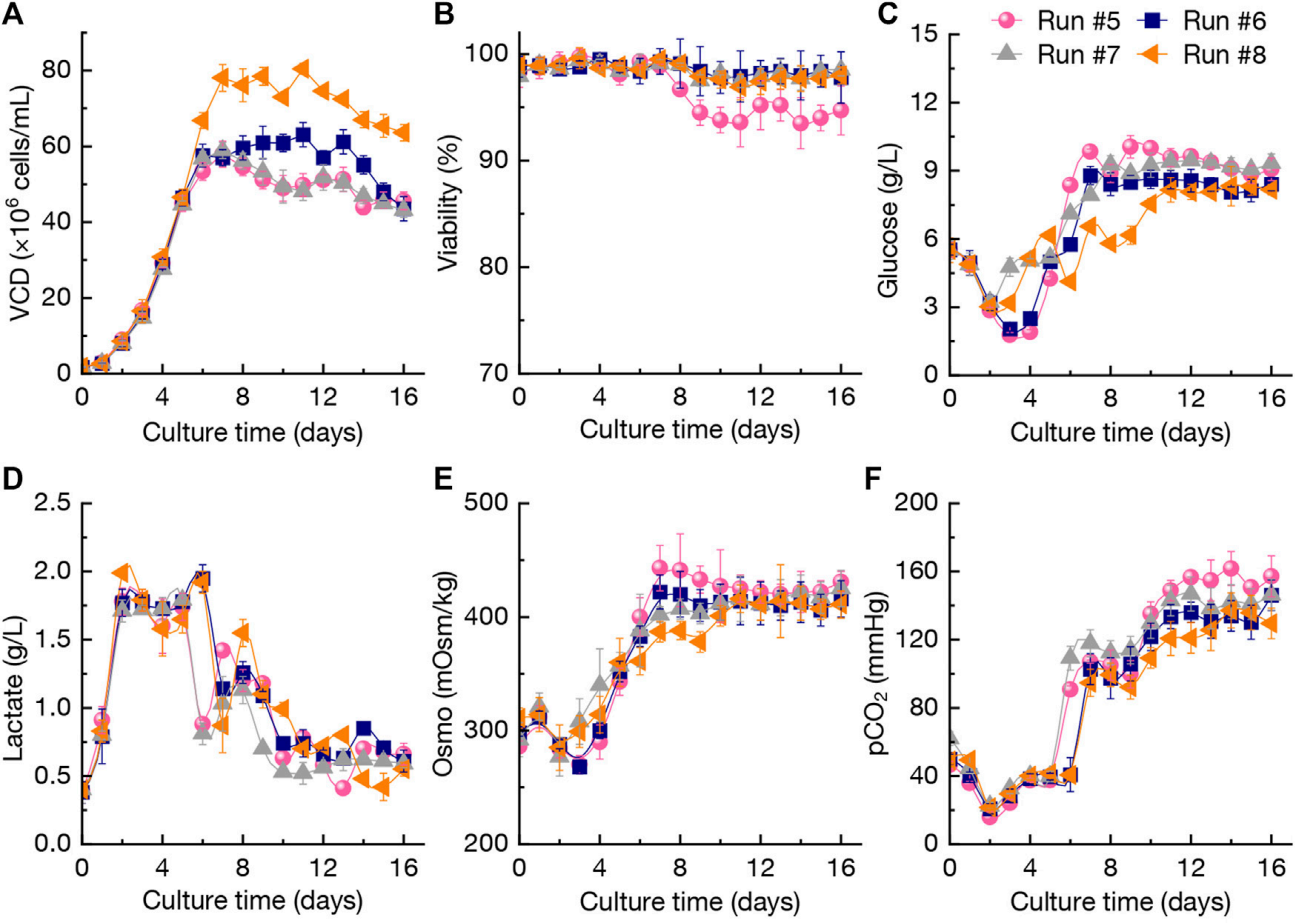
Changing profiles of VCD **(A)**, viability **(B)**, glucose concentration **(C)**, lactate concentration **(D)**, Osmo **(E)**, and pCO_2_ **(F)** during different perfusion cultures in 3-L bioreactor. The error bars indicate the SD of three independent experiments.

As shown in Figure 4A, the VCD of CHO cells during perfusion culture under N-2/N-1 adaption (run #5) reached 57.9×10^6^ cells/mL at day 7 and final VCD was 45.6×10^6^ cells/mL at day 16. In contrast, when reducing the culture temperature to 31.0°C from day 5 to day 6 (run #6), the VCD of CHO cells was higher than that of run #5 during the period of day 8–16. It was found that adjustment of the perfusion rates in day 2–6 (run #7) could not increase the VCD of CHO cells compared with runs #5-#6 (Figure 4A). In addition, increasing the perfusion rates (run #8) resulted in the highest VCD levels of 76.1 × 10^6^–80.5×10^6^ cells/mL at day 7–11. At the culture end at day 16, the final VCD still kept at a higher level of 63.6×10^6^ cells/mL. The viability of CHO cells in run #5 was the lowest level among the cultures after day 7, which indicated that N-2/N-1 adaption by BM-II could not improve the robustness of CHO cells growth. The viability of CHO cells in runs #5-#8 maintained at more than 95% without significant differences (*p* > 0.05, Figure 4B). The changing patterns of glucose concentration and lactate concentration in runs #5-#8 revealed that those two process parameters were not significantly influenced by different perfusion culture strategies, although the VCD and viability of CHO cells showed various trends (Figures 4C, D). Compared with run #5, the osmotic pressure in run #8 during day 6–16 with high perfusion rates kept relative lower levels (Figure 4E). Furthermore, the pCO_2_ in run #8 was also lower than that of run #5 (Figure 4F). Zhu et al. (2005) reported that elevating pCO_2_ from 50 mmHg to 150 mmHg under controlled osmolality leads to a 9% reduction in CHO cells growth rate with fed-batch culture, and a 60% reduction in cells growth was achieved when increasing the osmotic pressure from 316 mOsm/kg to 450 mOsm/kg (Zhu et al., 2005). The results obtained from the perfusion culture strategies (Figure 4) were consistent with the above conclusions.

**FIGURE 5:**
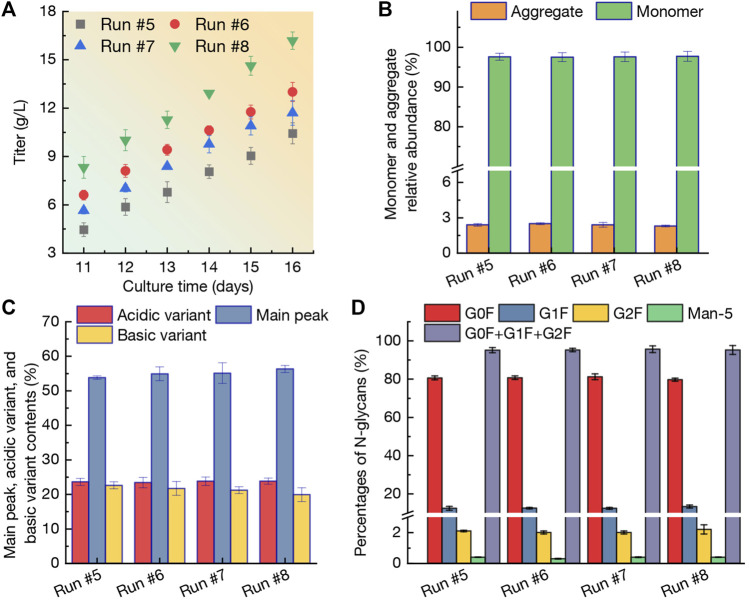
Profiles of mAb titer **(A)**, monomer and aggregate relative abundance **(B)**, variant contents **(C)**, and percentages of N-glycans **(D)** during perfusion cultures of run #5-#8. The error bars indicate the SD of three independent experiments.

The mAb production obtained from runs #5-#8 was shown in Figure 5A. At day 11, the titer reached 4.46 g/L in run #5, 6.61 g/L in run #6, 5.65 g/L in run #7, and 8.33 g/L in run #8, respectively. It is concluded that N-2/N-1 adaption method could restrict the CHO cells growth (Figure 4) and mAb production during culture process. The lower VCD and viability of CHO cells in run #5 (Figures 4A, B) resulted in the lowest titer. Furthermore, when using the high perfusion rate strategy (run #8), the titer at day 14 reached 12.93 g/L and final titer was up to a higher level of 16.19 g/L at day 16 (Figure 5A). In this case, the mAb production rate was 1.572 g/L/day which was significantly higher than other three runs (1.195 g/L/day for run #5, 1.278 g/L/day for run #6, and 1.210 g/L/day for run #7). Due to the vital role in therapeutic protein products, the aggregation of mAbs should be carefully investigated and analyzed (Wang 2005; Ha et al., 2022). Interestingly, as shown in Figure 5B, the relative abundance of monomer and aggregate maintained at similar levels of 2.3%–2.5% and 97.5%–97.7% in runs #5-#8 with no statistically significant difference (*p* > 0.05). The results revealed that the selected perfusion media (BM-II, FM-III, and FM-IV) could not affect the aggregation relative abundance significantly. The contents of main peak, along with the acidic and basic variants produced from the four perfusion runs were shown in Figure 5C. Since the variant of acidic and basic in mAbs are the heterologous structures formed under some modifications in the main peak (Ha et al., 2022). When adaption of CHO cells with BM-II before inoculation in bioreactor, the acidic variant and basic variant ratios were 23.6% and 22.6%. When reduction of the culture temperature at day 6, the corresponding indexes still kept similar levels of 23.4% and 21.7% (*p* > 0.05). When increasing the perfusion rate in run #8, the acidic variant also maintained 23.8% with *p* > 0.05, while the basic variant was reduced to a lower level of 19.9% (*p* < 0.05). Compared with the main peak in run #5 (53.8%), the contents of main peak was 54.9% in run #6 (*p* > 0.05), 55.1% in run #7 (*p* > 0.05), and 56.3% in run #8 (*p* < 0.05), respectively. Furthermore, the biological and physiological properties of proteins could be affected by N-glycans. As shown in Figure 5D, the percentage of G0F, G1F, and G2F in run #5 was 80.6%, 12.4%, and 2.1%, with a total N-glycans of 95.1%. Although the perfusion culture strategies varied in runs #6-#8, the N-glycans percentage was not significantly changed (95.2% for run #6, 95.5% for run #7, and 95.2% for run #8). In addition, the Man-5 ratio in the four perfusion cultures varied in a range of 0.3%–0.4% (Figure 5D). In summary, based on the results of mAb quality including titer, monomer and aggregate relative abundance, main peak and variants, and N-glycans percentages, it could be concluded that the culture strategy with higher perfusion rates (run #8) could achieve an excellent production of mAb with relative higher quality.

### 3.3 The mAb production performance under the optimized perfusion culture in 200-L bioreactor

Generally, it is considered that large-scale bioproduction is the key to a successful biopharmaceutical manufacturing. However, the mAb production performance under large-scale bioreactor was not desirable due to the lower cell viability and productivity (Gao et al., 2016; Vodopivec et al., 2019). Here, the effectiveness of the optimized perfusion culture strategy proposed in 3-L bioreactor (run #8) on the anti-PD-1 mAb production performance was further verified in the 200-L bioreactor (run #9).

**FIGURE 6:**
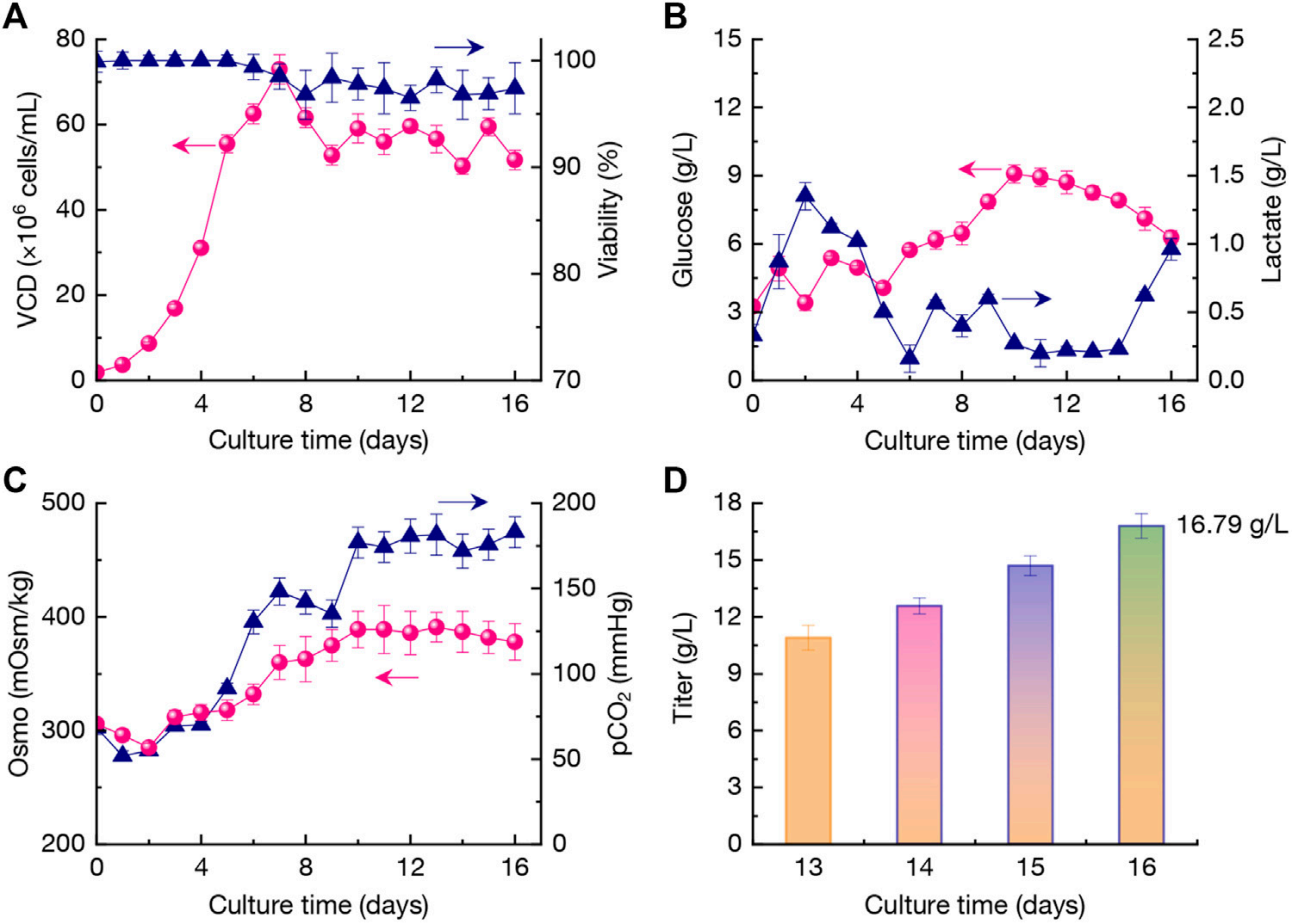
Changing profiles of VCD and viability **(A)**, glucose and lactate concentration **(B)**, Osmo and pCO_2_ **(C)**, and titer **(D)** during the optimized perfusion culture in a 200-L bioreactor. The error bars indicate the SD of three independent experiments.

As shown in Figure 6A, under the perfusion culture strategy, the VCD of CHO cells reached 55.5×10^6^ cells/mL at day 5 rapidly and then the cells growth rate was reduced gradually. The highest VCD reached 73.0×10^6^ cells/mL at day 7, which was lower than that of run #8 (80.5×10^6^ cells/mL at day 11, Figure 4A). The results indicated that larger bioreactor could affect the CHO cells growth mainly due to the various mixing environments. During the perfusion culture stage, the viability in run #9 maintained at higher levels of 96.5%–100%. The glucose concentration in culture broth was gradually increased from 3.25 g/L (day 0) to 9.08 g/L (day 10), and then it was reduced to a final concentration of 6.28 g/L at day 16 (Figure 6B). In this case, the glucose concentration during day 14–16 was lower than run #8 (Figure 4C). In addition, the lactate concentration maintained at lower levels of 0.20–0.27 g/L during day 10–14, which indicated that the assimilation of lactate by CHO cells was efficient. Afterwards, the accumulation of lactate was increased to a higher concentration of 0.96 g/L (Figure 6B). It has reported that pCO_2_ could accumulate to the higher levels in large-scale bioreactors when the formed CO_2_ from CHO cells was not removed efficiently (Zhu et al., 2005). As exhibited in Figure 6C, the pCO_2_ reached up to ∼175 mmHg after day 10 which was significantly higher than the culture performed in 3-L bioreactor (run #8, *p* < 0.05). It was mainly attributed to the higher lactate accumulation (Figure 6B). In addition, the osmolality under the perfusion culture strategy was kept at appropriate level of <390 mOsm/kg (Figure 6C), which might be beneficial for high-efficient mAb production.

The mAb production profiles were displayed in Figure 6D. At day 13, the titer was 10.89 g/L. The average productivity of mAb during day 13–16 reached 1.97 g/L/day with the higher final titer of 16.79 g/L. The titer was kept with comparable level with run #8 (16.19 g/L, *p* > 0.05). Furthermore, the overall production performances of different cultures including run #1, #2, #8, and #9 were summarized in Table 3. The relative abundant of aggregate in run #9 was 2.3% and the monomer ratio reached 97.6%. In this case, the contents of main peak, acidic variant, and basic variant were 56.3%, 23.5%, and 20.2%, respectively (Table 3). In addition, the total N-glycans of 96.5% was also obtained with 80.8% of G0F, 13.6% of G1F, and 2.1% of G2F, and the content of Man-5 was kept at a low level of 0.4%.

**TABLE 3:** Protein titers and relative abundant, mAb charge variant content, and percentages of N-glycans under different perfusion strategies.

Culture runs with perfusion strategies		Run #1 (3 L reactor)^a^	Run #2 (3 L reactor)^a^	Run #8 (3 L reactor)^b^	Run #9 (200 L reactor)^b^
Titer (g/L)	Day 14	8.73 ± 0.42	9.79 ± 0.52	12.93 ± 0.12	12.57 ± 0.42
	Day 16	N/A	N/A	16.19 ± 0.54	16.79 ± 0.65
Relative abundant (%)	Aggregate	2.2 ± 0.11	2.1 ± 0.08	2.3 ± 0.07	2.3 ± 0.11
	Monomer	97.8 ± 2.10	97.9 ± 1.10	97.6 ± 1.24	97.6 ± 0.89
Content (%)	Acidic variant	22.1 ± 0.97	24.2 ± 1.50	23.8 ± 0.90	23.5 ± 0.89
	Main peak	49.5 ± 2.90	55.4 ± 1.90	56.3 ± 1.00	56.3 ± 2.13
	Basic variant	28.4 ± 0.96	20.4 ± 1.96	19.9 ± 1.98	20.2 ± 1.20
N-glycans (%)	G0F	80.8 ± 1.30	80.4 ± 0.90	79.7 ± 0.80	80.8 ± 0.90
	G1F	12.5 ± 0.38	13.8 ± 0.40	13.3 ± 0.80	13.6 ± 0.32
	G2F	1.7 ± 0.08	2.2 ± 0.10	2.2 ± 0.29	2.1 ± 0.08
	G0F + G1F + G2F	94.9 ± 1.76	96.4 ± 1.40	95.2 ± 2.31	96.5 ± 1.30
	Man-5	0.5 ± 0.01	0.5 ± 0.02	0.4 ± 0.01	0.4 ± 0.01

N/A, not applicable for Run #1 and Run #2.

^a^ The parameters including relative abundant, content, and N-glycans were determined by the Day 14 samples in Run #1 and Run #2.

^b^ The parameters including relative abundant, content, and N-glycans were determined by the Day 16 samples in Run #8 and Run #9.

Perfusion culture processes have been widely used for enhancing the production performance by CHO or human cell lines. A CHO cell line expressing a humanized anti-CD52 mAb was cultivated in perfusion mode, which results in a higher productivity and stabilized product quality. In the perfusion process, the maximal volumetric productivity reached up to 0.55 g/L/day, which was 2.2-fold higher than that of the fed-batch culture (Zhuang et al., 2017). Recently, Schwarz et al. (2020) reported that the higher cell density of ∼80×10^6^ cells/mL could be achieved with perfusion cultivations and the recombinant human Erythropoietin (rhEPO) was stabilized expressed with a volumetric productivity of 0.6 g/L/day (Schwarz et al., 2020). Additionally, the recent advances of perfusion culture of CHO cells used in the biopharmaceutical industry with the demands for Industry 4.0 were also systematically reviewed in 2021 (MacDonald et al., 2021). In summary, the results obtained in present study revealed that the optimized perfusion culture strategy could high-efficient and stabilized produce anti-PD-1 mAb by CHO cells with a low raw materials cost, high titer/productivity, and stabilized quality (Table 3). It should be emphasized that, the effectiveness of the optimized perfusion culture strategy for other mAbs and rBPs production will be further investigated for process intensification.

## 4 Conclusion

In this study, the continuous perfusion culture was verified as an optimized method for mAb production by CHO cells with low-priced perfusion media. Then, the perfusion culture rates and culture temperature were also optimized for evaluation of mAb titer and quality in 3-L bioreactor. The results indicated that the product titer reached a higher level of 16.19 g/L when combining the high perfusion rates and delayed reduction of culture temperature at day 6. In addition, the effectiveness of the proposed perfusion culture strategy was successfully verified in a 200-L bioreactor, which produced 16.79 g/L mAb at day 16 and the product quality was also maintained. This study provides some guidance for the mAb production by CHO cells with process engineering methods.

## Data Availability

The original contributions presented in the study are included in the article/Supplementary Material, further inquiries can be directed to the corresponding author.
